# Factors influencing survival in sphingosine phosphate lyase insufficiency syndrome: a retrospective cross-sectional natural history study of 76 patients

**DOI:** 10.1186/s13023-024-03311-w

**Published:** 2024-09-27

**Authors:** Nancy Keller, Julian Midgley, Ehtesham Khalid, Harry Lesmana, Georgie Mathew, Christine Mincham, Norbert Teig, Zubair Khan, Indu Khosla, Sam Mehr, Tulay Guran, Kathrin Buder, Hong Xu, Khalid Alhasan, Gonul Buyukyilmaz, Nicole Weaver, Julie D. Saba

**Affiliations:** 1 Department of Pediatrics, University of California, San Francisco, CA USA; 2 Department of Nephrology, Alberta Children’s Hospital, Calgary, AB Canada; 3 Ochsner Clinical School, University of Queensland (Australia) and Ochsner Health, New Orleans, LA USA; 4 Center for Personalized Genetic Healthcare and Department of Pediatric Hematology/Oncology and BMT, Cleveland Clinic, Cleveland, OH USA; 5 Division of Pediatric Nephrology, Christian Medical College, Vellore, India; 6 Department of Nephrology, Perth Children’s Hospital, Perth, Australia; 7 Department of Neonatology and Pediatric Intensive Care, Ruhr-Universität Bochum, Bochum, Germany; 8 Department of Pediatrics, NAMO Medical Education and Research Institute, Shri Vinoba Bhave Civil Hospital, Silvassa, Dadra and Nagar Haveli, Daman and Diu India; 9 Department of Pediatric Pulmonology and Sleep Medicine, NH SRCC Hospital for Children, Mumbai, India; 10 Department of Immunology, Royal Children’s Hospital, Melbourne, Australia; 11 Department of Pediatric Endocrinology and Diabetes, Marmara University School of Medicine, Istanbul, Turkey; 12 Pediatric Nephrology Department, University Children’s Hospital Zurich, Steinwiesstrasse 75, 8032 Zurich, Switzerland; 13 Department of General Pediatrics and Hematology/Oncology, University Hospital Tuebingen, University Children’s Hospital, Hoppe-Seyler-Strasse 1, 72076 Tuebingen, Germany; 14 Department of Nephrology, Children’s Hospital of Fudan University, National Pediatric Medical Center of China, Shanghai, China; 15 Department of Pediatrics, College of Medicine, King Saud University, Riyadh, Saudi Arabia; 16 Department of Pediatric Endocrinology, Ankara City Hospital, Ankara, Turkey; 17 Department of Pediatrics, Cincinnati Children’s Hospital Medical Center, Cincinnati, OH USA

**Keywords:** SPLIS, *SGPL1*, Inborn error of metabolism, Nephrotic syndrome, Adrenal insufficiency, Kidney transplantation, Vitamin B6, Pyridoxal 5′-phosphate, Gene therapy

## Abstract

**Background:**

Sphingosine-1-phosphate lyase insufficiency syndrome (SPLIS) is a recently recognized inborn error of metabolism associated with steroid-resistant nephrotic syndrome as well as adrenal insufficiency and immunological, neurological, and skin manifestations. SPLIS is caused by inactivating mutations in *SGPL1*, encoding the pyridoxal 5’phosphate-dependent enzyme sphingosine-1-phosphate lyase, which catalyzes the final step of sphingolipid metabolism. Some SPLIS patients have undergone kidney transplantation, and others have been treated with vitamin B6 supplementation. In addition, targeted therapies including gene therapy are in preclinical development. In anticipation of clinical trials, it will be essential to characterize the full spectrum and natural history of SPLIS. We performed a retrospective analysis of 76 patients in whom the diagnosis of SPLIS was established in a proband with at least one suggestive finding and biallelic *SGPL1* variants identified by molecular genetic testing. The main objective of the study was to identify factors influencing survival in SPLIS subjects.

**Results:**

Overall survival at last report was 50%. Major influences on survival included: (1) age and organ involvement at first presentation; (2) receiving a kidney transplant, and (3) *SGPL1* genotype. Among 48 SPLIS patients with nephropathy who had not received a kidney transplant, two clinical subgroups were distinguished. Of children diagnosed with SPLIS nephropathy before age one (n = 30), less than 30% were alive 2 years after diagnosis, and 17% were living at last report. Among those diagnosed at or after age one (n = 18), ~ 70% were alive 2 years after diagnosis, and 72% were living at time of last report. SPLIS patients homozygous for the SPL R222Q variant survived longer compared to patients with other genotypes. Kidney transplantation significantly extended survival outcomes.

**Conclusion:**

Our results demonstrate that SPLIS is a phenotypically heterogeneous condition. We find that patients diagnosed with SPLIS nephropathy in the first year of life and patients presenting with prenatal findings represent two high-risk subgroups, whereas patients harboring the R222Q SGPL1 variant fare better than the rest. Time to progression from onset of proteinuria to end stage kidney disease varies from less than one month to five years, and kidney transplantation may be lifesaving.

## Background

Sphingosine-1-phosphate lyase insufficiency syndrome (also known as SPLIS, renal, endocrine, neurological and immune (RENI) syndrome, *SGPL1* deficiency, nephrotic syndrome type 14, NPHS14, steroid-resistant nephrotic syndrome type 14, and familial steroid-resistant nephrotic syndrome with adrenal insufficiency) is an inborn error of metabolism (OMIM **#** 617575) associated with kidney, endocrine, immunological, neurological, and skin manifestations [1]. These manifestations have been described in detail in several recent review articles [2–6]. SPLIS is caused by inactivating mutations in *SGPL1* (OMIM 603729), which encodes sphingosine-1-phosphate lyase (SPL) (UNIPROT #O95470), a pyridoxal 5’-phosphate dependent enzyme that catalyzes the final step of sphingolipid metabolism [7]. Specifically, SPL is an integral membrane protein of the endoplasmic reticulum membrane that catalyzes the irreversible cleavage of phosphorylated sphingoid bases including sphingosine-1-phosphate (S1P), a bioactive sphingolipid and the final product of sphingolipid metabolism [8]. In so doing, SPL generates two products, ethanolamine phosphate and hexadecenal. SPL guards the only exit point of sphingolipid metabolism, and as such it serves an essential function. The pathophysiology of SPLIS is complex and likely involves contributions from substrate accumulation and aberrant signaling through S1P receptors, loss of biochemical products needed for autophagic flux and other cell functions, and accumulation of cytotoxic sphingolipid intermediates including ceramides. In that regard, some patients with SPLIS have been shown to exhibit elevated levels of plasma S1P and other sphingolipids, which are being developed as disease biomarkers in SPLIS [9, 10]. SPL activity contributes to global lipid homeostasis within the liver and cholesterol homeostasis in cells and tissues, as shown by the high lipid and cholesterol levels of *Sgpl1* knockout mice [11, 12]. SPL promotes flux from sphingolipid metabolism to glycerophospholipids [13, 14]. The role of SPL in autophagy and proteostasis may be important in promoting the removal of aggregate-prone proteins, particularly within neurons [15–17]. SPL regulates calcium homeostasis and the steady state levels of sphingolipid intermediates, both of which have been implicated in skin biology and its water barrier function in health and in sphingolipid disorders including SPLIS [18–21].

Data from SPLIS patients in whom family genetic studies were performed indicate that SPLIS is always caused by recessive inheritance of bi-allelic germline mutations in *SGPL1*. The *SGPL1* gene contains 15 exons, with the ATG start site in exon 2. Variants associated with the SPLIS condition have been found in almost every exon, with the preponderance of variants occurring in the highly conserved central pyridoxal 5′-phosphate-binding domain [22]. Indels, truncations, splice site variants and missense substitutions in *SGPL1* have been observed in SPLIS patients. The biochemical consequences of these mutations range from a total lack of *SGPL1* mRNA production, to *SGPL1* mRNA being produced but resulting in little or no expression of SGPL1p (likely due to protein misfolding as is typical of inborn errors of metabolism), to gene and protein expression at wild type levels but with reduced enzyme activity, likely due to reduced binding affinity for substrate and/or cofactor.

The first reports of the monogenic cause of this condition in 2017 focused on two of the most common clinical features, namely steroid-resistant nephrotic syndrome (often associated with focal segmental glomerulosclerosis pathology), and primary adrenal insufficiency [23, 24]. Adrenal insufficiency may include low glucocorticoids with or without low mineralocorticoids, and in boys may also be associated with gonadal dysgenesis and low testosterone levels [6]. Other effects observed in SPLIS patients include peripheral neuropathy, central nervous system involvement, immune dysfunction, hypercholesterolemia, and ichthyosis or acanthosis [3, 10, 23, 24]. Nervous system signs of SPLIS include strabismus, squint, deafness, and lower limb weakness, and these may be associated with characteristic basal ganglia findings on magnetic resonance imaging of the brain [25, 26]. Immunodeficiency is signaled by absolute lymphopenia with or without other immune deficiencies and frequent infections [3]. Failure to thrive is also a common SPLIS feature. Less frequent findings include anemia, cardiac anomalies, hypothyroidism, brain anomalies, vomiting, diarrhea, food intolerance, and retinal lesions. Aside from lymphopenia and anemia, laboratory findings may include proteinuria, hypoalbuminemia, hypertriglyceridemia, increased ACTH, and increased plasma sphingolipids. Radiological studies may reveal adrenal calcifications, renal abnormalities, ascites, hydrops, increased cortical bone, and T2 flair, atrophy, and corpus callosum defects on brain MRI. Human phenotype ontology cites 36 term associations for *SGPL1* (NCBIGene:8879): adrenal insufficiency, ataxia, autosomal recessive inheritance, childhood onset, cryptorchidism, developmental regression, diffuse mesangial sclerosis, edema, focal segmental glomerulosclerosis, generalized hypotonia, global developmental delay, hyperpigmentation of the skin, hypertriglyceridemia, hypoalbuminemia, hypoglycemia, hypogonadism, hypothyroidism, ichthyosis, infantile onset, juvenile onset, lymphopenia, mental deterioration, mesangial hypercellularity, microcephaly, micropenis, nephrotic syndrome, peripheral neuropathy, podocyte foot process effacement, progressive, proteinuria, ptosis, seizure, sensorineural hearing impairment, stage 5 chronic kidney disease, steroid-resistant nephrotic syndrome, strabismus [27].

Current treatment for SPLIS is largely supportive and includes hormone supplementation, kidney replacement therapy, intravenous immunoglobulins, transfusions, physical therapy, and nutritional support. Targeted therapeutic strategies to address the root cause of the condition are being explored. Gene therapy using an adeno-associated viral vector to deliver a healthy human *SGPL1* gene to SPLIS patients is in preclinical development [12, 28]. Cofactor supplementation with pyridoxine has been shown to improve SPL function and reduce sphingolipids in some SPLIS patient-derived fibroblasts, and there is anecdotal evidence of clinical benefit in some patients who harbor missense *SGPL1* mutations [10]. Based on these promising findings, clinical trials in SPLIS are on the horizon.

In anticipation of clinical trials, it will be essential to characterize the natural history of SPLIS. Evaluation of the various influences on disease severity and survival can help identify subsets of patients with different outcome risks. This may aid in clinical trial design and patient stratification. Natural history studies can additionally provide a historical control group for clinical trials in rare diseases with high mortality rates, wherein a randomized control group may not be ethically appropriate [29, 30]. This study focused specifically on identifying clinical and genetic factors that influence survival outcomes in SPLIS patients. Toward that end, we performed a retrospective analysis of 76 SPLIS patients in whom the diagnosis of SPLIS was established in a proband with at least one suggestive finding and biallelic *SGPL1* variants identified by molecular genetic testing and for whom sufficient data were available for analysis, e.g., genotype, number of cases reported per year, country of origin, follow up of at least 6 months for living patients. Due to their highly complex nature, immunological profiling, blood plasma sphingolipid profiling, and other paraclinical endpoints were not considered in this study and will be addressed comprehensively in separate reports. Our overall results demonstrate that SPLIS is phenotypically heterogeneous, and that patients diagnosed with SPLIS nephropathy in the first year of life represent a high-risk subgroup for which the risk/benefit of transplantation and/or gene therapy may be appropriate.

## Methods

### Study design

We conducted a retrospective cross-sectional analysis of survival data and related factors in 76 SPLIS patients reported or recruited between 2017 and 2023 from USA, Canada, Argentina, Brazil, United Kingdom, Spain, France, Germany, Turkey, Afghanistan, Iran, Iraq, Pakistan, Saudi Arabia, Morocco, Singapore, Peru, Serbia, China, India, Gambia and Australia. Case data was taken from published work and from personal communications with treating physicians as shown in Table 1. The present study was conducted at the University of California San Francisco in the Department of Pediatrics. It was performed by reviewing available data from published reports, seeking follow up information regarding the reported patients, and obtaining primary data from health care providers caring for reported and unreported SPLIS patients. Health care providers contributed patient data with the informed consent of the subjects or their patients if subjects were less than 18 years of age. This study was posted on the ClinicalTrials.gov website as: “Sphingosine phosphate lyase insufficiency syndrome- Observational study and patient registry (International) recruiting – NCT04885179”.

**Table 1:** Method of patient identification (HCP = health care provider)

Method of identification	# of Cases
First reports (Lovric et al., Prasad et al.)	26
Sibs/relatives of cases in first reports	8
Reported by others (2018–2023)	21
Letter to pediatric subspecialties	2
**HCP contact us, reported in Zhao et al	7
HCP contact us, not previously reported	9
Data analytics, not previously reported	1
Scientific interactions, not previously reported	2

### Data collection

Data were collected from published reports and/or questionnaires and medical records for unpublished cases. Not all data types were available for all patients. For all results presented, the number of patients for whom the relevant data were available and included in the analysis is stated within the Table or Figure legend. Types of data include patient *SGPL1* genotype, parental *SGPL1* genotype, patient gender, family history of SPLIS or SPLIS-related symptoms, country of residence, country of origin, age of the patient, organ/system involvement, age of the patient at first sign of SPLIS, first detection of proteinuria, time from proteinuria to end stage kidney disease (ESKD), first detection of endocrine defect, first sign of neurological impairment, first sign of immunological impairment, age at last report, age of death, and cause of death. Detailed analyses of immunological profiles, plasma sphingolipids, and other paraclinical parameters were not included in the current study. For the fourteen new cases presented in this study, laboratory tests including urine protein levels, growth parameters, and hematological and immunological parameters were considered abnormal if they fell outside of the age-specific pediatric reference ranges provided by the local medical center. In most cases, published reports did not cite local laboratory reference values. Results reported as abnormal by authors were accepted prima facie as being abnormal in relation to the laboratory standard for age. When quantitative results of urine protein levels were available for analysis, proteinuria was defined as ≥ 100 mg protein/m^2^/day or ≥ 0.5 mg protein/mg creatinine (up to age 2) and ≥ 0.2 beyond age 2 [31]. However, in all patients in whom quantitative urine protein levels were available, proteinuria was in the nephrotic range (i.e., ≥ 2 mg protein/mg creatinine). For end-stage kidney disease, the definition of a glomerular filtration rate of less than 15 ml/min/1.72 m^2^ was used.

### Inclusion criteria

Study subjects included living and deceased subjects anywhere in the world at any age from prenatal diagnosis forward who received a genetic diagnosis of SPLIS defined as biallelic variants in *SGPL1* (including pathogenic variants, likely pathogenic variants, and variant of unknown significance) and at least one reported clinical feature of SPLIS.

### Exclusion criteria

(1) No genotype reported, (2) no country of origin reported; (3) no year of case report available.

### Ethical approval

The University of California San Francisco Institutional Review Board (IRB) approved the study. Written informed consent for the acquisition of genetic, molecular, and clinical data was obtained from the parents of the enrolled patients except in cases wherein the information was published and only data from published reports were used for information collection. Please, refer to the Declarations section for institutional review board and consent information.

### Genetic testing

Testing was performed by the reporting health care provider using commercial or institutional genetic diagnostic whole exome or whole genome sequence analysis or by a genetic diagnostic panel including the *SGPL1* gene.

### Variant calling

When a variant was present in the ClinVar database, the designation of pathogenic, likely pathogenic, or uncertain significance provided in ClinVar was used. For variants not present in ClinVar, American College of Medical Genetics criteria were applied and the results provided [32]. In addition, Varsome tool was used to garner in silico predictions for each of the variants not listed in ClinVar [33]. Results are provided in Table 4.

### Statistical methods

Subject characterizations, including demographics, were analyzed as categorical or continuous variables collected in the study. Categorical variables were analyzed by Fisher’s exact test. Continuous variables were analyzed as normally distributed (parametric) or not normally distributed (nonparametric). Continuous variables were reported as mean with standard variation, or as median and interquartile range (IQR). Welch’s, or Bonferroni corrections were applied as necessary. Two-tailed t-test was used for normally distributed data; Mann–Whitney was used for nonparametric data. The limit of statistical significance is set to *p* < 0.05. Probability of survival was measured by Kaplan–Meier. The log-rank Mantel-Cox was used to compare survival between patient groups.

## Results

### Demographics of SPLIS

**Fig. 1:**
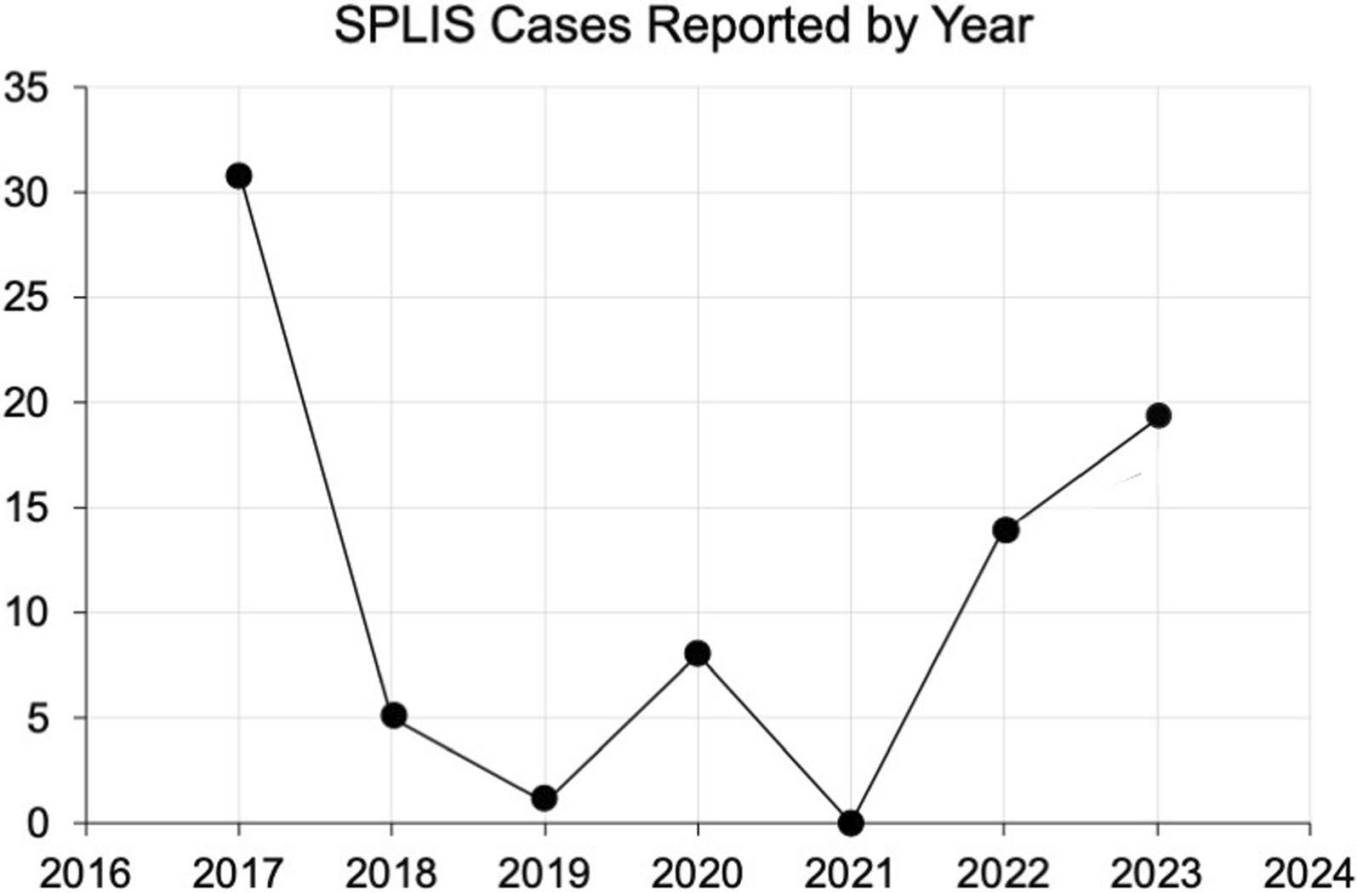
SPLIS cases reported by year. Number of cases reported each year starting with the initial two reports linking biallelic genetic variants of *SGPL1* with a syndrome characterized by steroid-resistant nephrotic syndrome and primary adrenal insufficiency by Lovric et al. [23] and Prasad et al. [24]. Cases in 2023 include the 14 new cases in this study not previously reported

SPLIS was first recognized as a monogenic cause of steroid resistant nephrotic syndrome and primary adrenal insufficiency in 2017 [23, 24]. We recently reported the estimated worldwide prevalence of SPLIS to be approximately 11,000 [34]. However, the number of recognized cases is only a fraction of that, likely due to lack of awareness about this new condition. The number of cases reported/identified per year since 2017 is shown in Fig. 1.

**Fig. 2:**
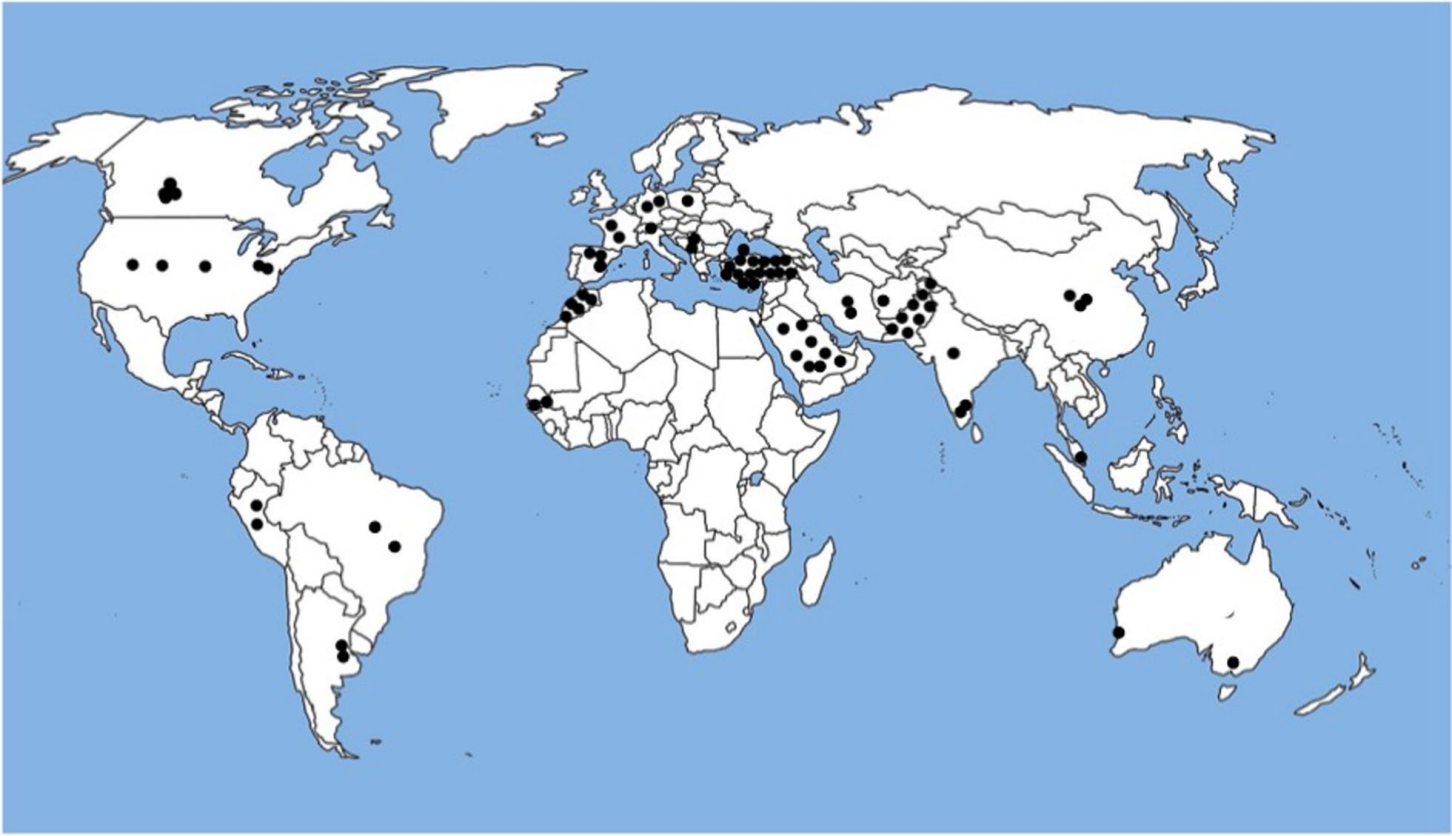
Geographic distribution of SPLIS cases/ancestries in this study. Each black dot represents an individual SPLIS case within the country designated; marked locations are not specific for city or region

The SPLIS cases included in our study come from a variety of sources including the initial two reports that revealed inactivating *SGPL1* mutations as the likely underlying cause of the condition [23, 24], and additional publications reporting one or more cases of genetically confirmed SPLIS [3–6, 9, 10, 22, 35–49]. We are also aware of multiple cases of genetically confirmed SPLIS through personal contact with researchers or health care providers of patients described in this study and not previously reported. In total, 76 patients with genetically confirmed SPLIS are known to us and serve as the baseline SPLIS patient census. In Methods and Materials, the breakdown of our source of patients is given in Table 1. The geographic and ethnic origin of these patients were wide-ranging, including every continent with the exception of Antarctica (Fig. 2). However, the cases tended to cluster in geographic regions where consanguinity rates and inbreeding are higher than average as a result of cultural norms, including Pakistan, Middle East/North Africa, and the Hutterite colonies of southern Canada.

### Clinical features of SPLIS

The most common clinical features in 76 SPLIS patients are shown in Table 2, and the age of onset of the three most prominent features (nephropathy, adrenal insufficiency and neuropathy) is shown in Table 3. Approximately half of the known SPLIS patients are still alive to our knowledge, and half are confirmed deceased. The male:female sex distribution is 1.1–1. Although most reported patients were diagnosed within the first few years of life, age at first presentation varied widely from the prenatal period, with patients presenting with prenatal findings to the oldest age of presentation being 15 years in a patient with isolated peripheral neuropathy. Kidney disease is usually the first finding and frequently occurs coincidentally with either adrenal or neuronal abnormalities, as shown in Table 3.

**Table 2:** Clinical features of SPLIS

SPLIS features	# or %
# of cases in census	76
Male/Female	1.1/1
# of *SGPL1* variants	45
# of ethnicities/nationalities	23
Still living	50%
Prenatal diagnosis or findings	22%
Consanguinity reported	61%
Family history	56%
Presented in first year of life	54%
Median age of death in deceased (age range)	0.6 years (0–8.6 years)
Prenatal findings & survive 3 years	0
Kidney involvement	78%
End stage kidney disease (ESKD)	35%
Neurological defect	43%
Primary adrenal insufficiency	63%
Hypothyroidism	33%
Lymphopenia	30%

**Table 3:** Organ involvement at first presentation of SPLIS

Organ	Number with 1st sign at ≤ 1 yr	Number with 1st sign at 1–10 yrs	Number with 1st sign at ≥ 11 yrs
Kidney	32	**20**	3
Adrenal	15^a^	**5**	0
Neurological	15^a^	6	2

^a^11 coincident with kidney involvement

### SPLIS-associated SPL variants

All cases of SPLIS known to us involve the recessive inheritance of biallelic *SGPL1* variants either in homozygous or compound heterozygous state, with all parents of probands being asymptomatic heterozygous carriers when parental genetic testing information was available.

**Fig. 3:**
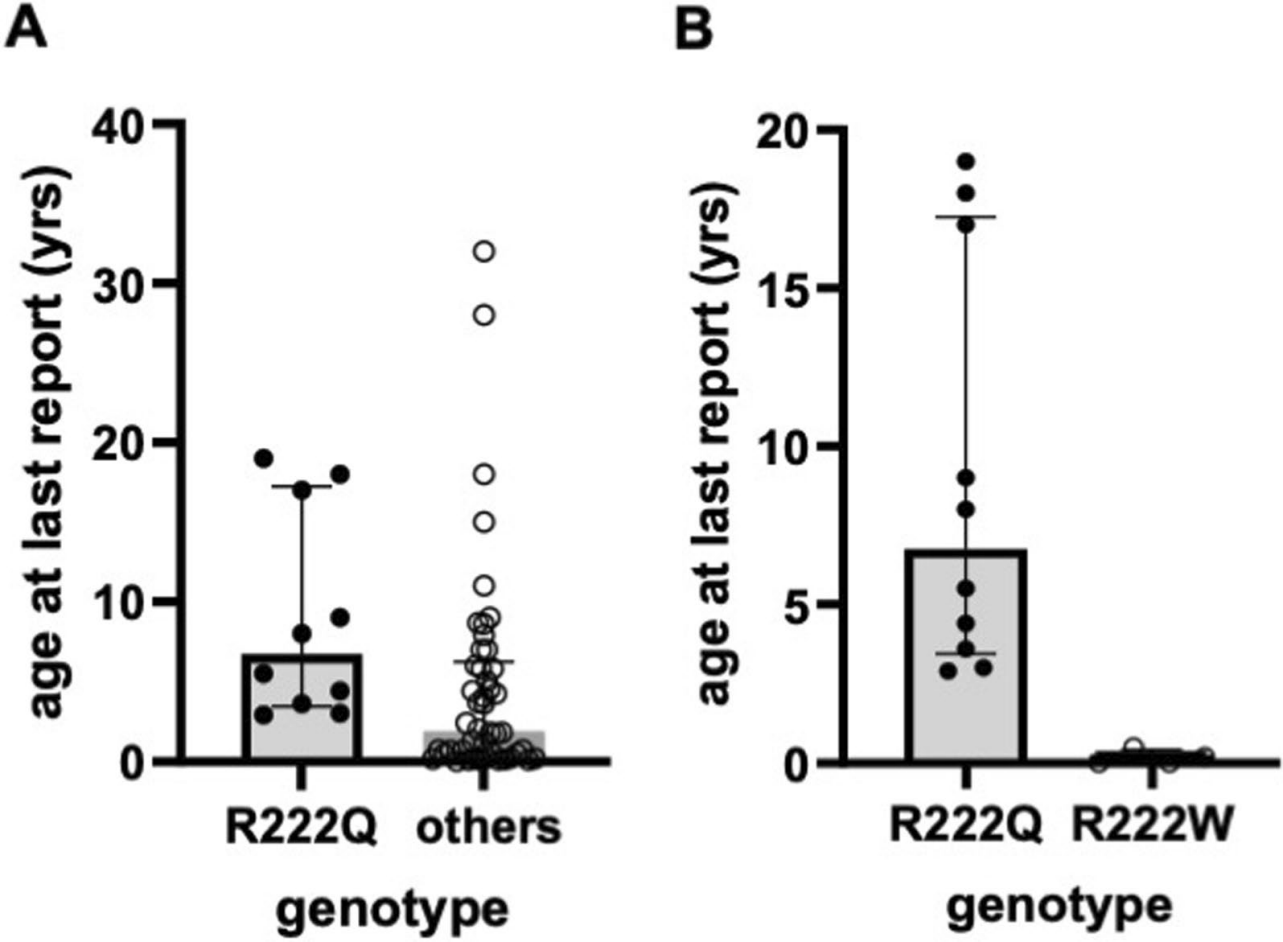
Longevity of R222Q patients compared to other variants. There were 13 patients homozygous for the R222Q variant: three were not included in the analysis because they were transplanted. Thus, there were 10 R222Q patients in the analysis. The patients with other genotypes numbered 49, excluding transplants. There were four R222W patients. **A** The median age at last report was significantly greater in the R222Q group: 6.8 years (IQR = 13.8) vs. all others 1.7 (IQR = 5.6) (*p* = 0.004, Mann–Whitney with Bonferroni correction). **B** The R222Q group also had greater survival compared to the R222W group, for whom the median age at last report was 0.1 years (IQR = 0.4). The *p* value of R222Q vs. R222W was 0.004 (Mann Whitney with Bonferroni correction)

In total, 44 different genotypes have been identified in the 76 SPLIS patients in this study (Table 4). Of these, slightly more than half were found in homozygous state, and the remainder were found in association with 1 or more other variants. Twenty-one variants were predicted to result in complete loss of function (pLOF) due to either catastrophic effect on mRNA stability and/or protein synthesis, or substitution of the critical cofactor-binding Lysine at amino acid position 353 (Table 4). Not counting survivors who received a kidney transplant, of the 21 patients harboring two pLOF variants, only 28% were still living at the time of last report. Comparing survival in subjects with bi-allelic pLOF variants versus all other subjects, no significant difference was observed. Five missense variants (R222Q, Y416C, R340W, S346I, and R222W) accounted for 75% of all reported SPLIS cases and had the following median age (range) in years at time of last report: R222Q = 8.0 (2.9–19); Y416C = 6.2 (1.7–12); R340W = 3.67 (1.25–5.7); S346I = 0.1 (0–0.5); R222W = 0.09 (0–0.42). The most prevalent variant was the R222Q SPL variant, which was always in the homozygous state. The R222Q variant represented 29% of all reported variants, 17% of all reported SPLIS cases, and was associated with a wide range of phenotypes (Table 5), with 77% still living at the time of last report (including those receiving kidney transplants) and 80% if transplanted subjects are excluded in the calculation (Table 5). Five of the thirteen R222Q patients did not exhibit kidney involvement at the time of this report. In contrast, of non-transplanted SPLIS patients with variants other than R222Q, 41% were alive at the time of last report. Excluding those receiving a kidney transplant, median age of R222Q patients at time of last report was 6.8, significantly older than the median age of 1.7 for the rest of the cohort (Fig. 3A). (Refer to Fig. 3 legend for additional statistical details). Patients with homozygous R222Q variants were of Turkish, Saudi and Pakistani ancestries.

**Table 4:** SPLIS-associated *SGPL1* variants and survival outcomes

	SPLIS-associated *SGPL1* variants^a,b^	ClinVar designation (ACMG criteria)	Fraction alive at last report	Notes	References
1	R222Q	Pathogenic	11/13	2 alive were transplanted	[5, 6, 22, 24], this study
2	R340W	PP1, PP4, PP3: likely pathogenic	3/6	1 alive was transplanted	[18, 19, 24, 32], this study
3	Y416C	Likely pathogenic	5/6	1 heterozygous with S202L; 1 heterozygous with D315Y	[5, 8], this study
4	S346I	Pathogenic	0/5		[5]
5	R222W	Pathogenic	0/4		[5]
6	Y15C	PP4	3/3	1 homozygote; 2 sibs with 3 variants each including Y15C, K353E and M409R	[8, 25]
7	S202L	PP4	2/3	1 alive transplanted; 1 heterozygous with A316T; 1 heterozygous with Y416C	[5, 8, 28]
8	R278Gfs*17^b^	PVS1, PP4, PP1: pathogenic	2/2	2 heterozygous with E312G (mutation leads to nonsense-mediated RNA decay)	[5]
9	S3Kfs*11^b^	Pathogenic	2/3		[5, 6]
10	E132G^b^	PM3, PP1, PP4, PS3: pathogenic	2/2	Mutation leads to nonsense mediated RNA decay; 2 heterozygous with R278fs*17	[5]
11	I184T	Uncertain significance PM3, PP1, PP4	2/2	2 heterozygous with S361*	[16]
12	S361*^b^	PVS1, PP1, PP4: pathogenic	2/2	2 heterozygous with I184T	[16]
13	A316T	Uncertain significance	2/2	1 alive transplanted; 1 heterozygous with S202L	[5, 27, 34] (27 and 34 refer to same patient)
14	S65Rfs*6^b^	Pathogenic	2/2 0/2	2 homozygous living 2 heterozygous with c.1298 + 6 T > C	[6] [30]
15	F545del^b^	Pathogenic	0/2		[6, 24]
16	G360V	Uncertain significance	1/2		[8, 23, 24]
17	K353E^b^	PP3, PP4, PM1 Uncertain significance	2/2	cofactor binding site; 2 sibs with 3 variants each including Y15C, K353E and M409R	[25]
18	M409R	PP3, PP4 Uncertain significance	2/2	2 sibs with 3 variants each including Y15C, K353E and M409R	[25]
19	L173Q	PP3, PP4, PP1 Uncertain significance	1/2		[32], this study
20	R505*^b^	Pathogenic	0/2		[17]
21	c.1298+6T>C^b^	Pathogenic	0/2	2 heterozygous with S65Rfs*6	[30]
22	c.1566+2T>C^b^	PVS1, PP4: Uncertain significance	0/1	1 heterozygous with C285Y	[8]
23	C285Y	PP3, PP4, PM3: Uncertain significance	0/1	1 heterozygous with c.1566 + 2 T > C	[8]
24	L312Ffs*30^b^	Pathogenic	1/1		[17]
25	G35Afs*49^b^	PVS1, PP4, PM3 Pathogenic	0/1	1 heterozygous with K353R (cofactor binding site)	[8]
26	K353R^b^	PP3, PM3, PM1, PP4: Likely pathogenic	0/1	1 heterozygous with G360Afs*49	[8]
27	L173Pfs*55(;)(?)^b^	Pathogenic	0/1		This study
28	Q289Tfs*12^b^	PVS1, PP4, PM3: pathogenic	0/1		This study
29	S362T	PP3, PP4	0/1		This study
30	W45X	Pathogenic	0/1	1 heterozygous with S240I	This study
31	S240I	PP3, PM3, PP4: Uncertain significance	0/1	1 heterozygous with W45X	This study
32	F411Lfs*56^b^	PVS1, PP4, PM3: pathogenic	0/1		[20]
33	D315Y	PP3, PP4: Uncertain significance	1/1	1 heterozygous with Y416C	This study
34	Y331^^b^	Pathogenic	0/1	1 heterozygous with F290L	[8, 35]
35	P311R	PP3, PP4: Uncertain significance	0/1		[27, 34]
36	D350G	PP3, PP4: Uncertain significance	1/1		[29]
37	Q239fs*8^b^	PVS1, PP4, PM3: Pathogenic	0/1		[24]
38	E142Rfs*20^b^	PVS1, PP4, PM3: Pathogenic	0/1		This study
39	Q478E	PP3, PP4: Uncertain significance	1/1		[33]
40	F290L	likely pathogenic	0/1	1 heterozygous with Y331^	[8, 35]
41	D5Tfs*Ter8^b^	PVS1, PM3, PP4: pathogenic	1/1		This study
42	G343R	PP3, PP4: uncertain significance	0/1		[31]
43	N171D	PP4: Uncertain significance	0/1		[21]
44	c.1_27del; Start loss^b^	PVS1, PP4, PM3: pathogenic	0/1		[26]

^a^Variants are homozygous unless otherwise stated

^b^Predicted loss of function

Varsome in silico predictions were obtained for each of the variants not present in the ClinVar database. The following The following Varsome definitions were applied:

PP1—multiple affected siblings with same combination of variants

PP3—multiple lines of computational evidence support a deleterious effect on the gene or gene product

PP4—patient phenotype or family history is highly specific for a disease with a single genetic etiology

PVS1—LOF and splice variants

PM3—recessive disorders, detected in trans with a pathogenic variant

PS3—well established in vitro or in vivo functional study supportive of a damaging effect on gene or gene product

PM1—located in mutational hot spot and/or critical and well-established functional domain

**Table 5:** Characteristics of SPLIS patients with SPL R222Q variant

Case	Age (yr) at 1st sign of kidney disease	Age (yr) at sign of adrenal insufficiency	Age (yr) at sign of skin abnormality	Age (yr) at sign of neurological abnormality	Age (yr) at last report	Living (L) or deceased (Dc) at last report
1	19	6.5			19	L
2*	3	3			8	L
3	2	2			2.9	Dc
4*	2.5		0.7		5	L
5		0.5	0.5		8	L
6		0.5	0.5		4	L
7		1.5	1.5		3.6	L
8		2			5.5	L
9	17	15		15	17	L
10	6		0 (at birth)		9	L
11**	6	1			8	Dc
12				8	18	L
13	2.7	0.6			4.4	Dc

^*^Cases 2 and 4 had transplants at age 8 and 5, respectively

^**^Case 11 had a transplant at age 7; graft rejection and death at age 8

Two other variants, Y416C and R340W, comprised 13% of variants and 8% of reported SPLIS cases. The Y416C variant was found in homozygous state in 4 patients of the North American Hutterite colonies and heterozygous in 2 patients from Singapore and the USA. Overall survival of these combined patients with Y416C variants was 66.7%. SPLIS patients with the (always homozygous) R340W variant were from Brazil, Turkey and India, and their overall survival at the time of last report was 50%. The S346I variant representing 11% of variants and 7% of SPLIS cases was found in homozygous state in one Moroccan family, and no case survived (Table 6). In contrast to the generally more favorable outcome of SPLIS patients homozygous for the R222Q variant, no patients homozygous for R222W variant survived (Table 6 and Fig. 3B.) This variant represented 9% of all variants and 5% of cases, all being of Turkish ancestry. SPLIS patients with all other variants taken together exhibited an overall survival at time of last report of 48%, with patients harboring indels having 30% survival. SPL is a pyridoxal 5′-phosphate dependent enzyme, and the cofactor binds at lysine 353. Two variants at K353 were found in SPLIS patients and can be presumed to be complete loss of function variants, as shown by in vitro experiments [50]. A patient with a K353R variant did not survive, consistent with the known inactivation of SPL by K353R substitution. One pair of siblings each harboring 3 *SGPL1* variants (Y15C, K353E and M409R) are alive at age 4 and 7 at time of last report. Based on the high prevalence of variant Y15C [34], it is likely benign. Based on the predicted loss of function K353E, and the likely benignity of Y15C, the M409R variant might provide some residual SPL function.

**Table 6:** Characteristics of individuals with homozygous R222W and S346I variant SPLIS

Case	Age at 1st sign of kidney disease	Age at sign of adrenal insufficiency	Age at sign of skin abnormality	Age at sign of neurological abnormality	Age at last report	Living (L) or deceased (Dc) at last report
R222W						
1	0.25	0.25		0.42	0.42	Dc
2	0.17				0.17	Dc (fetal hydrops)
3						Fetal demise
4						Fetal demise
S346I						
1	0.0		0.0	0.0	0.5	Dc
2	0.1		0.1	0.1	0.1	Dc
3						Fetal demise
4						Fetal demise
5	0.1				0.25	Dc

### Causes of death in SPLIS

Many of the clinical features of SPLIS have a profound impact on quality of life, are associated with significant morbidity, and have the potential to impact mortality. End-stage kidney disease (ESKD) if left untreated is lethal. Dialysis or kidney transplantation interventions may be lifesaving but are associated with risks of infection, surgical complications, and tissue rejection. SPLIS patients often exhibit variable degrees of immunosuppression, predisposing them to infection from viral, bacterial and other pathogens. Food intolerance issues and failure to thrive may further reduce nutritional status and risk of infection in SPLIS children. Seizures, which may be associated with adrenal insufficiency or neurological involvement in SPLIS patients, can have catastrophic consequences. Cardiac involvement with heart failure has been reported in several cases of SPLIS, increasing susceptibility to cardiorespiratory events. To determine which of these many factors were the most catastrophic to SPLIS patients, we assessed the reported causes of death and number or percent of patients in each category among the 30 cases for whom this information is available. The results are given in Table 7. ESKD with or without failure-to-thrive (FTT) or complications of kidney transplantation was the main cause of death and accounted for 30% of deaths. Sepsis or infection represented the second most frequent cause of death, accounting for 20% of deaths. The remainder of deaths were reported to be caused by cardiovascular complications, cerebral edema, fetal demise, sudden death, or unspecified cause.

**Table 7:** Causes of death in SPLIS

Cause of death	# of cases
ESKD ± FTT	9
Sepsis/infection	6
Cardiorespiratory failure	4
Fetal demise with heart failure	2
Fetal demise (unknown)	2
Sudden death	2
Post-transplant	1
Cerebral edema	1
Congestive heart failure/Arrhythmia	1
Hypovolemic shock	1
Not specified	1

### Outcome of SPLIS patients with kidney disease

A group of 48 SPLIS patients with kidney involvement who did not receive a transplant were analyzed for their survival outcomes defined by age at last report. These patients were selected based on: (1) having received no more than supportive care (specifically, including patients receiving transfusions, dialysis, physical therapy, steroids, and other non-targeted medications and (2) the availability of at least 6 months follow up after diagnosis; (3) age at last report as well as age at diagnosis of SPLIS signs—kidney disease, neurological defects, or adrenal insufficiency. Our analysis demonstrated that two clinical subgroups of SPLIS patients with kidney disease exhibited significantly different survival patterns. Mortality prior to age 5 was 81% in patients (n = 30) who presented with kidney involvement in the age range of at birth to less than one year who had not received a kidney transplant within the first year of life. This high mortality contrasts with the 27% mortality prior to age 5 among 18 patients with kidney involvement as a 1st sign of SPLIS from age one or more years. Thus, older age at first sign of SPLIS as kidney disease improves survival.

**Fig. 4:**
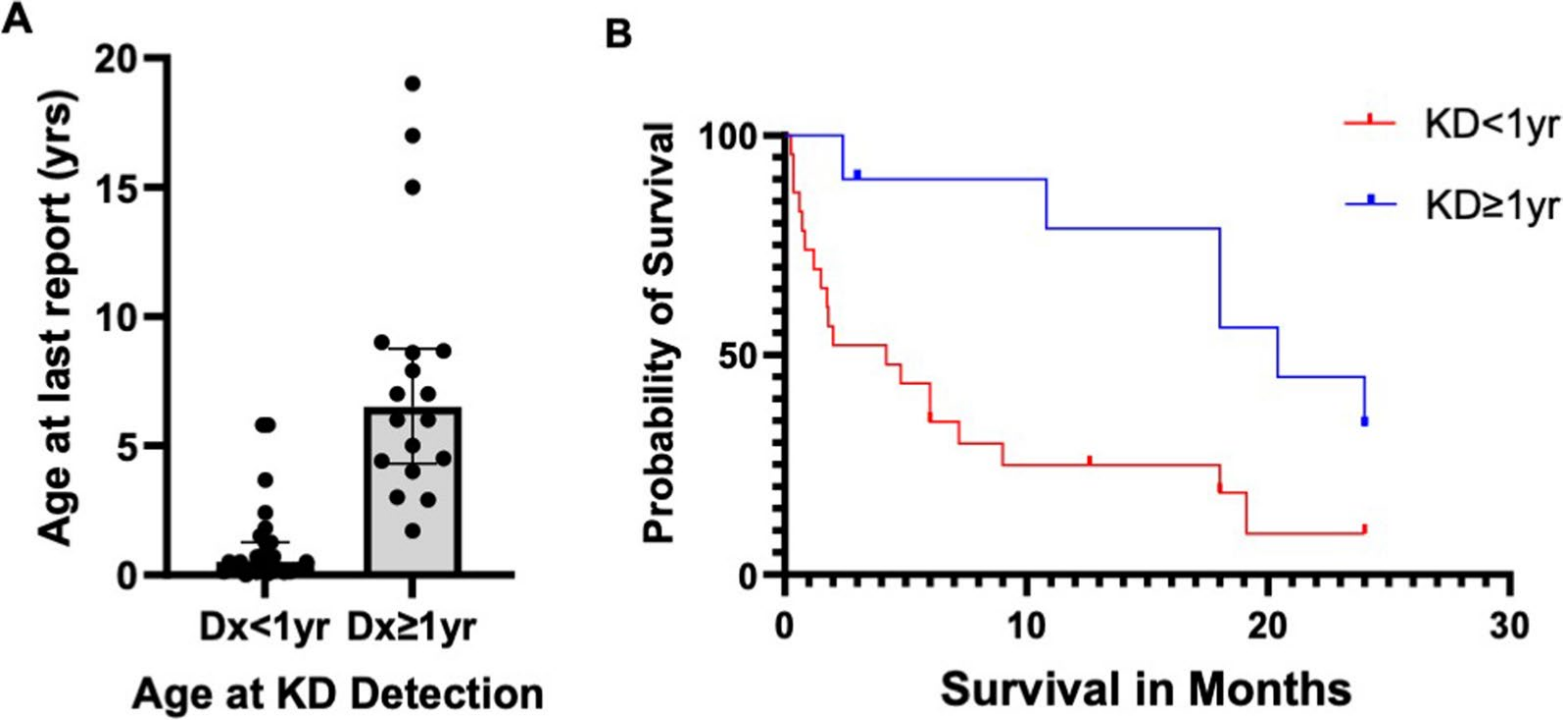
Survival outcomes in two subgroups of SPLIS patients. **A** Age at last report as a measure of survival was compared in the group of patients diagnosed with kidney disease in infancy (< 1 year) compared to those who presented with kidney disease beyond infancy (≥ 1 year). The data include patients whose first sign of SPLIS was not KD, but they do not include patients who received a kidney transplant. Thirty patients meeting these inclusion and exclusion criteria were diagnosed before age 1; 18 patients were diagnosed at or after their first birthday. The median (IQR) age at last report for the Dx < 1 group was 0.4 (0.8); the median (IQR) for the Dx ≥ 1 was 6.5 (4.6). The difference between medians was statistically significant, *p* < 0.0001). **B** Probability of survival of these same two groups of patients during two years of study. The median survival is 3.0 months for the infant group and 20.4 months for the older group of patients. The difference in the survival curves is significant (*p* = 0.0019; log-rank (Mantel–Cox test)

Figure 4 shows the difference in survival outcomes in two SPLIS subgroups, namely those in whom the diagnosis of kidney disease was made prior to one year of age, and those in whom kidney involvement was detected at equal to or greater than one year. Figure 4A shows the survival outcome as the age in years at last report, and Fig. 4B shows the survival times during the first two years of the study in months after the onset of proteinuria or kidney involvement. The median (IQR) age at last report of 30 non-transplanted SPLIS patients diagnosed before age one year was 0.4 (0.8) years compared to a median of 6.5 (4.6) years for 18 non-transplanted patients diagnosed at age one or later. Only 5 (17%) of the 30 patients diagnosed before age one were living at last report. In contrast, 12 (67%) of the patients diagnosed at or after age one were alive at last report. The difference between the two medians is statistically significant at *p* < 0.0001 (Mann–Whitney). Table 8 shows a comparison of the age at first sign of SPLIS, age at last report, and percent survival for SPLIS patients separated by presence or absence of kidney disease, age at onset of kidney disease, and transplant status.

**Table 8:** Outcomes based on kidney involvement, age of involvement and treatment

Group	Patient group description	Age at 1st sign of SPLIS median (IQR)	Age at last report median (IQR)	Patient survival
1	Non-kidney disease + Transplanted KD	1.0 (1.8) Group 1 vs. Group 2 *p* = 0.0503	8 (11.5) Group 1 vs. Group 2 *p* = 0.0001	15 of 17 (88%)
2	All KD Except Transplanted	0.45 (2.7)	1.38 (5.6)	20 of 48 (42%)
3	KD onset < 1y Except Transplanted	0.11 (0.25) Group 3 vs. Group 4 *p* < 0.0001	0.38 (0.8) Group 3 vs. Group 4 *p* < 0.0001	7 of 30 (23%)
4	KD onset ≥ 1y Except Transplanted)	3.0 (3.4)	6.5 (4.6)	13 of 18 (72%)
5	KD onset ≥ 1y + non-kidney disease + Transplanted KD	2.0 (4.0) Group 3 vs. Group 5 *p* < 0.0001	7 (4.6) Group 3 vs. Group 5 *p* < 0.0001	28 of 35 (80%)

### SPLIS nephrosis progresses rapidly, and kidney transplantation is life-saving

Onset of kidney disease (proteinuria) ranged from birth to 22 years of age. In many patients with SPLIS and steroid-resistant nephrotic syndrome, kidney involvement was present at or before birth. In patients who presented with proteinuria without ESKD, the time from first detection of proteinuria to ESKD varied from less than one month to 60 months. The median time to progression was 30 months. Table 9 shows the rate of kidney disease progression to ESKD for 21 subjects in whom sufficient data were available for analysis. Of the patients who were diagnosed with KD at age one year or more, all 6 progressed to ESKD from 24 to 60 months after the diagnosis of kidney disease. Among those diagnosed with kidney disease in infancy, 8 progressed within 6 months, and 7 progressed more slowly, reaching ESKD at between 11 and 60 months. The data sets are small, and further observation would be required to confirm the reproducibility and import of these results.

**Table 9:** Months from diagnosis of kidney disease (Dx KD) to end stage kidney disease (ESKD)

Variants	Age Dx KD (yrs)	Months to ESKD	Transplant	pLOF/PV	Immunodeficiency
R505*	0.02	0.25	No	Yes	No mention
R505*	0	3	No	Yes	No mention
G360Afs*49; K353R	0.1	1.5	No	Yes	Yes
G360V	0.2	6	No	No	Yes
c.1566+2T>C; C285Y	0	1	No	Yes	Yes
F290L;Y331*	0	4	No	Yes	Yes
Q289fs*	0	1.5	No	Yes	Yes
Y416C	0.2	3	No	No	Yes
S65Rfs*6	0.9	11	Yes	Yes	No mention
R340W	0.3	36	Yes	No	Yes
S3Kfs*11	0.8	50	Yes	Yes	No mention
S3Kfs*11	0.5	54	Yes	Yes	Yes
R340W	0.5	23	No	No	No
S65Rfs*6 c.1298+6T>C	0.9	59	No	Yes	No
S65Rfs*6 c.1298+6T>C	0.8	60	No	Yes	No
c.395A>G; R278fs*17	1	36	No	Yes	No mention
R222Q	2.5	30	Yes	No	No
R222Q	3	60	Yes	No	No mention
S202L; Y416C	3	24	No	No	Yes
R222Q	6	36	No	No	Yes
S202L; A316T	18	48	Yes	No	Yes

**Fig. 5:**
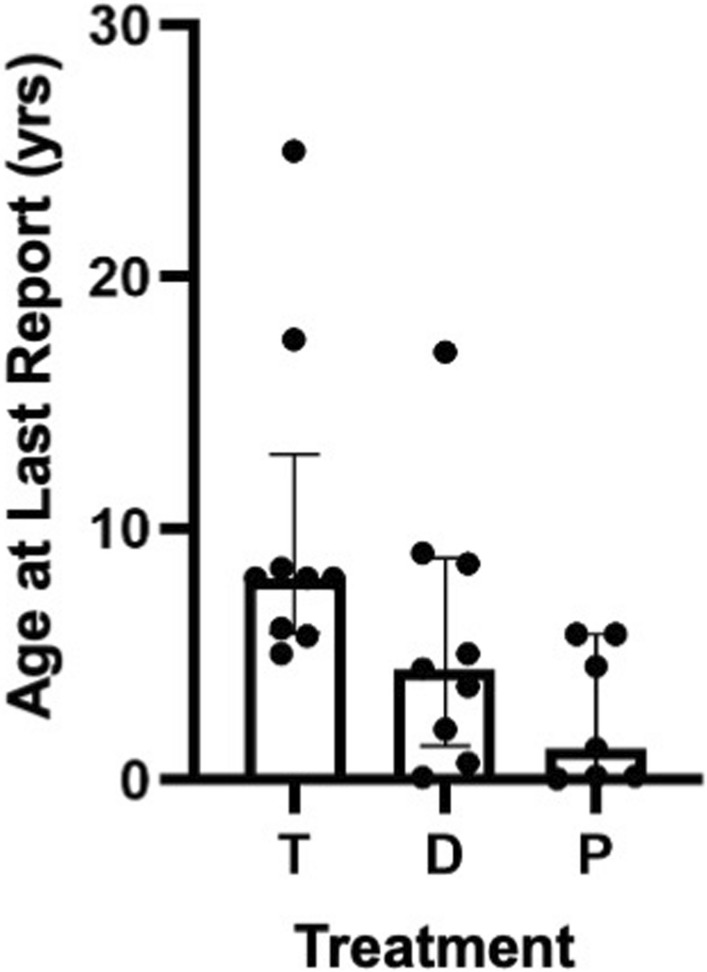
Impact of treatment on survival in SPLIS patients with nephropathy. Treatment of SPLIS patients varied: 9 received a kidney transplant (T); 9 only received dialysis (D); 7 only received palliative care (P). It should be noted that one of the patients receiving a transplant also received pyridoxine therapy. Outcomes with dialysis were not significantly better than palliative care (*p* > 0.05). In contrast, transplanted patients were older at time of last report than those receiving palliative treatment (*p* = 0.004); Mann Whitney with Bonferroni correction. Median among transplanted cases was 8 years (IQR 7); dialysis 4.4 years (IQR 7.4); palliative care 1.3 years (5.6). Statistically no significant difference was found in age at last report between T and D cases (*p* > 0.05), although survival trended higher in the T cases compared to D cases

Once in ESKD, patients either received palliative intervention, dialysis, or kidney transplantation. A group of 7 patients who received palliative treatment, 9 who received dialysis, and 9 who received a kidney transplant had the survival results shown in Fig. 5. When survival was compared in the three treatment groups, the median (IQR) age at last report was 1.3 (5.6) for the group receiving palliative care, 4.4 (7.4) for the group receiving only dialysis, and 8 (7) for the transplanted group. Based on our results, kidney transplantation afforded a significant benefit to SPLIS patients compared to palliative care, whereas dialysis did not improve survival significantly over palliative care.

### Outcome of SPLIS patients without renal involvement

Eight SPLIS patients in our cohort did not have onset of renal involvement as of the time of last report, two of whom were diagnosed before age 1. All are living at last report (although we have no follow up on one of the two infants). Four of the patients had isolated neurological findings, three had isolated primary adrenal insufficiency, and one had both. Age at diagnosis ranged from 0.17 to 12 years (median 1.75, IQR 7.8). Median age at last report for this group was 6.7 years (IQR 22.6), ranging from 0.17 to 32 years.

### Outcome of SPLIS patients with prenatal findings

**Fig. 6:**
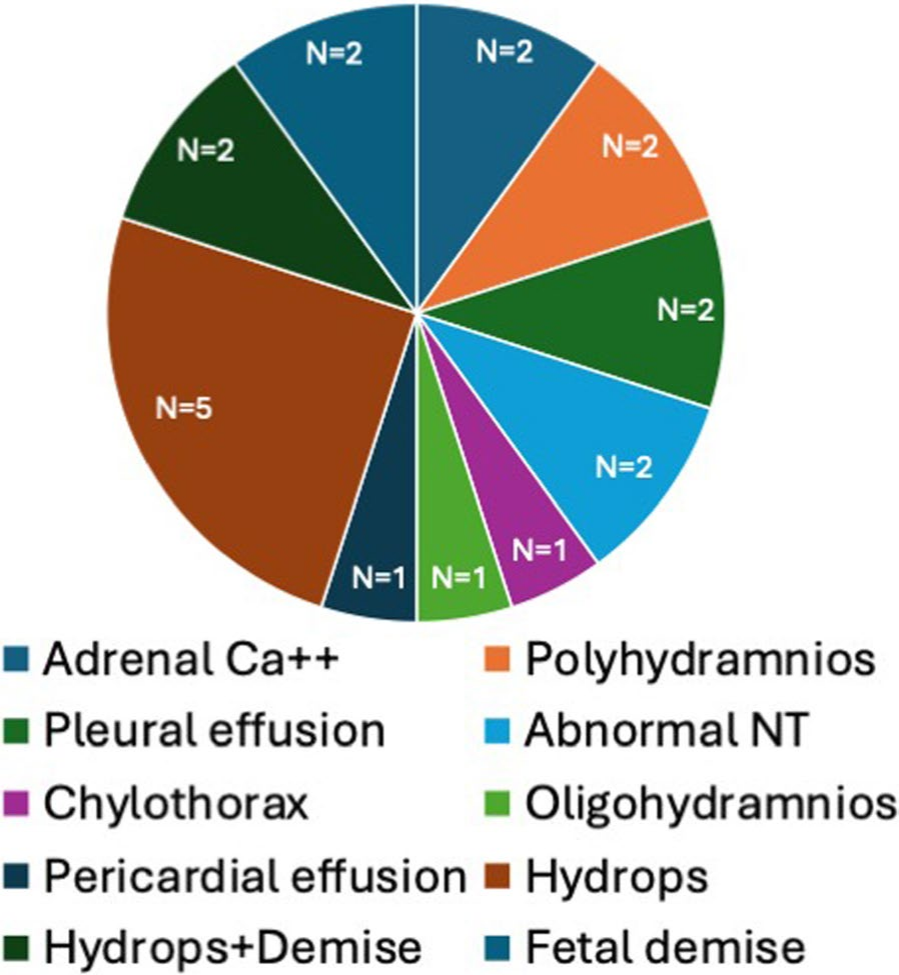
Prenatal findings in SPLIS. The figure depicts the type and relative proportion of prenatal findings among all prenatal findings in SPLIS patients for whom the data were available. Ca^++^, calcification; NT, nuchal translucency

A variety of prenatal findings have been described in SPLIS patients (Fig. 6). These include hydrops, adrenal calcifications, oligohydramnios, skin edema, polyhydramnios, adrenal hemorrhage, nuchal translucency, brain developmental defects, and fetal demise. Regardless of the type of finding, whenever prenatal abnormalities were detected, they portended the early demise of the patient.

### Outcome of SPLIS patients by gender

Gender distribution was nearly equal among SPLIS subjects. There was a greater number of surviving males than females, with 58% of males living at time of last report, median age 3 (IQR 7.3) compared to 37% of females, median age 2.2 (IQR 6.1), although this difference was not significant (*p* = 0.09). More males than females had received a kidney transplant (8 of 38 males vs. 1 of 35 females), and more males that females had the more favorable *SGPL1* R222Q variant genotype (10 of 38 males vs. 3 of 35 females). These two factors likely account for the higher survival of males compared to females in our cohort, but more observations will be required to confirm this finding.

## Discussion

Due to the recent discovery of SPLIS, the natural history of the condition is still poorly understood. In this retrospective cross-sectional study, information from 76 cases was used to characterize the natural progression of the disease, identify clinical subgroups, and establish influences on patient survival. Several important trends were observed.

First, SPLIS is a highly heterogeneous condition, as is typical of many inborn errors of metabolism. Heterogeneity is observed in SPLIS with respect to age of first presentation, severity, organ involvement, and outcomes. Age at first clinical symptoms varied from in utero (prenatal findings) to late childhood/teenage years. In some cases, the condition led to catastrophic events in infancy (seizures, developmental regression, ESKD, and terminal events), whereas others are living in their fourth decade. Some patients exhibited only peripheral neuropathy, whereas others (usually infants) exhibited all the characteristic features including kidney, endocrine, neurological, immunological and skin manifestations. This degree of heterogeneity presents challenges for predicting the disease course, counseling families, and designing and conducting clinical trials. To aid in future clinical trial study design and endpoint selection, we sought to identify the most common disease manifestations, any clinical subgroups, and factors correlating with survival as an outcome.

Other than lymphopenia, which may be clinically significant but is often found incidentally, kidney disease is the most common manifestation of SPLIS and the most likely to lead to death in early childhood. With a few exceptions, patients with signs of SPLIS before age one year had kidney disease. Once recognized, proteinuria progressed to ESKD within five years in SPLIS patients. This is consistent with the established patterns of progression in pediatric steroid-resistant nephrotic syndrome, wherein 50% of cases progress to ESKD within ten years, but with progression being more likely and more rapid (i.e., within 4–5 years) in children diagnosed with monogenic forms of the disease [51]. Many of the infants with nephropathy progressed to ESKD within less than six months. However, there was no clear pattern that predicted rapid progression in this group.

Patients presenting with kidney disease as a first sign of SPLIS before the age of one year usually did not survive beyond age one in the absence of treatment. Adrenal insufficiency and skin abnormalities (usually ichthyosis) sometimes manifest before one year of life. Eleven patients had kidney disease and adrenal insufficiency before age one year. The presence or absence of adrenal insufficiency in patients with SPLIS nephropathy did not influence survival outcomes (data not shown). Patients who progressed to kidney failure before the age of one were more likely to have immune deficiency. Considering infection and sepsis represented the second most frequent cause of death in SPLIS patients, a more detailed characterization of the immunological function in SPLIS patients and establishing whether prophylaxis with antibiotics, intravenous immunoglobulin, or other proactive measures impact outcome are warranted. This information will be essential to elucidating the clinical consequences of lymphopenia in SPLIS and guiding standard of care.

Other influences on survival in SPLIS included the presence of prenatal findings, which was uniformly associated with early mortality. On the other extreme, patients homozygous for the SPL R222Q variant fared better than patients with all other genotypes combined. It has been shown that this variant may be responsive to cofactor supplementation [10]. We have found that pyridoxine supplementation can prevent SPLIS nephrosis in a knock-in mouse model of SPL R222Q homozygous variant SPLIS (our unpublished data). This suggests that patients with the R222Q genotype may be appropriate candidates for a trial of pyridoxine therapy.

There is good evidence showing an association of genotype with phenotype in SPLIS patients including and in addition to the encouraging outcome associated with the R222Q variant. As previously reported, mutations can destabilize the SPL protein and thus weaken or even eliminate enzymatic activity, thus promoting disease [10, 23, 52]. Some mutations such as R222Q are responsible for a less severe form of the disease, whereas other mutations such as R222W and S346I are calculated to cause a much greater destabilization of the protein and a more rapidly developing disease with a catastrophic outcome. In the most severe cases of SPLIS, kidney disease generally manifests before age one, and survival may be less than one year after diagnosis. On the other extreme, some individuals with SPLIS are living productive lives in their twenties and thirties. To quantitate and compare the effects of genotype on SPL stability and thus effectiveness in metabolizing S1P, we used the age at last report as a measure of survival after SPLIS diagnosis. Patients diagnosed with signs of SPLIS before the age of one year had a median age at last report of 0.4 years compared to patients diagnosed at or after age one year, whose median age at last report was 6.5 years. Kidney disease in the R222Q set of cases developed only after age one year, or not at all during this study. The median survival of SPLIS patients with the R222Q genotype (6.8 years, IQR 13.8) is significantly greater than that of either R222W or S346I genotype. The median for R222W is 0.1 (IQR 0.4) and for S346I is 0.1 (IQR 0.38). Two cases in each of the R222W and the S346I patient groups suffered fetal demise.

We should note that the 222 position in each of the two chains of the SPL dimer is intimately involved in the stability of the catalytic site. The substitution of glutamine for arginine (R222Q) breaks hydrogen bonds that help maintain the integrity of the catalytic site [23]. The more severe disease caused by R222W is likely due to not only disruption of critical hydrogen bonds at the 222 position, but also due to steric clash caused by substitution of the bulky tryptophan substitution for the wild type arginine [52]. Steric clash combined with disruption of hydrogen bonding should cause further distortion of the molecular conformation in the catalytic site resulting in severe loss of enzyme activity. However, even the bulky amino acid substitution of isoleucine for serine in a position remoted from the catalytic site, 346, also can cause destabilizing perturbations that lead to loss of enzyme activity, as shown by S346I cases [23]. The substitution of isoleucine for serine destabilizes the protein to an extent that is estimated to be 10 times that caused by substitution of glutamine for arginine [23]. While these genotype/phenotype correlations suggest that the degree of residual enzyme activity determines disease severity, systematic in vitro analysis of the enzyme activity of purified variant proteins compared to wild type enzyme will be necessary to confirm this.

The number of patients receiving a kidney transplant in our study was small. Nonetheless, we observed a significant therapeutic effect of transplantation in SPLIS patients with kidney disease, compared to patients receiving either dialysis or palliative care. There remains a theoretical concern that high levels of circulating sphingolipids in SPLIS patients could ultimately damage the donor kidney over time. However, we have no evidence in support of this notion. Future studies conducted in rodent models of SPLIS may be informative in addressing this question. In the meantime, the mechanism of kidney damage has not yet been determined, and the survival benefit to SPLIS patients with kidney failure is clear. Considering the fact that definitive treatments are already in development and clinical trials may be on the horizon, kidney transplantation would seem an appropriate life-extending intervention in SPLIS patients with ESKD. We speculate that a donor kidney could potentially provide a source of SPL enzyme activity and thereby reduce the risk of kidney and non-kidney SPLIS manifestations and post-transplant kidney failure. Future studies may clarify if this is the case or not. The current study did not gather information on the post-transplant experience in SPLIS. Addressing this complex topic adequately would involve consideration of patient age, *SGPL1* genotype, native kidney pathology, pre- and post-transplant immunosuppressive regimens, type and HLA/ABO compatibility of graft, other medications, other SPLIS features and the impact of transplantation on various clinical and quality-of-life endpoints. Thus, we elected not to include this topic in the current study. However, a detailed investigation of the post-transplant experience in SPLIS is clearly needed.

The non-uniform method of data collection and inconsistent types of data available for our cohort was a limitation of our study. Due to the rarity of SPLIS, the high mortality rate, and the recent recognition of this diagnostic entity, only a limited number of patients were available for data collection. Thus, we opted for inclusivity, using all available data from published reports and personal communications, at the cost of creating a non-uniform data set.

An additional limitation of our study was the exclusion of immunological, biochemical, radiological and other paraclinical results in our analysis. Immunological profiling and plasma sphingolipid analyses are highlty complex endeavors, each of which involves detailed methodology, establishment of pediatric reference range values, and quantitation of many different parameters. These endpoints are being comprehensively analyzed under two ongoing clinical studies and will be reported separately. Imagenology results were also not included in the current study. The limited reports and description of radiological results available in the current cohort of SPLIS patients was consistent with our previously reported description of brain magnetic resonance imaging, brain computed tomography, and abdominal ultrasound and computed tomography findings in a small series of SPLIS patients [26]. However, the lack of radiological results in the current study may also be considered a limitation.

Our overall results provide an initial survey of the spectrum of SPLIS manifestations and identify major influences on overall survival in SPLIS patients. The current study represents the largest study of SPLIS patients to date, with 76 subjects, 14 of whom are newly reported cases—the largest single cohort to date. The spectrum of presentations and severity of disease in our previously unreported cohort and in the study as a whole are consistent with those described in several previous reviews of the literature including our own (Pournasiri et al., Choi et al., Weaver et al.), and those of Tran et al., Roa-Bautista et al., Ozturk et al., Yang et al., and Maharaj et al. These other reports focused on skin, endocrine and immunological manifestations of SPLIS. Our study provides novel information by revealing factors affecting survival including kidney involvement, age of proteinuria detection, time to progression to ESKD, genotype, and presence of prenatal findings. Our findings should be helpful in counseling families. Our study should also help to guide the design of a SPLIS prospective natural history study that could validate these findings and provide a practical historical control group for future SPLIS clinical trials. In the future, comprehensive analyses of plasma biomarkers, immunological parameters, radiological findings, and the biochemical and clinical impact of currently available interventions (i.e., kidney transplantation, vitamin B6 supplementation) in SPLIS patients should shed more light on the clinical features and management of SPLIS. Sufficient understanding of the disease will support the need of gene replacement treatment in many patients.

## Conclusions

Our results corroborate previous studies demonstrating that SPLIS is a phenotypically heterogeneous inborn error of sphingolipid metabolism. We report fourteen new cases of SPLIS. We show that patients diagnosed with SPLIS nephropathy in the first year of life represent a high-risk subgroup with a mortality of approximately 80% for which the risk/benefit of kidney transplantation and/or gene therapy may be appropriate. Prenatal findings portend a dismal outcome in SPLIS. Genotype/phenotype interactions are beginning to emerge, with patients homozygous for the SPL R222Q variant representing a subgroup of SPLIS with longer overall survival than all other genotypes combined. Future studies comparing the level of residual enzyme activity in different SGPL1p variants are needed to confirm the relationships between genotype and phenotype. Time to progression to ESKD in SPLIS is between one month and five years. Kidney transplantation affords improved outcome compared to dialysis or palliative care in SPLIS patients with ESKD. Future studies characterizing the post-transplant experience in SPLIS in greater detail will be helpful in guiding treatment decisions. A prospective natural history study could further substantiate these findings and provide a practical historical control group for future SPLIS clinical trials.

## Data Availability

All data supporting the findings of this study are available within the paper. SPLIS-associated genotypes and references in which the data were extracted for each subject are provided in Table 4. Example from reference 5 (Lovric et al.): https://www.jci.org/articles/view/89626.

## Abbreviations

ACTH: Adrenocorticotropic hormone
CI: Confidence interval
Dc: Deceased
D: Dialysis
ESKD: End stage kidney disease
FTT: Failure to thrive
HCP: Health care provider
IQR: Interquartile range
KD: Kidney disease
L: Living
NT: Nuchal translucency
P: Palliative
pLOF: Predicted loss of function
S1P: Sphingosine-1-phosphate
SPL: Sphingosine phosphate lyase
SPLIS: Sphingosine phosphate lyase insufficiency syndrome
yr: Year
